# Dihydroxypropyl Chitosan: A Biorenewable Platform for the Design of Novel Fabric Care Additives

**DOI:** 10.1021/acs.iecr.4c03632

**Published:** 2024-11-27

**Authors:** Marcellino D’Avino, Ruth Chilton, Gang Si, Mark R. Sivik, David A. Fulton

**Affiliations:** † Chemistry-School of Natural and Environmental Sciences, Newcastle University, Newcastle Upon Tyne NE1 8QB, U.K.; ‡ Newcastle Innovation Centre, The Procter & Gamble Company, Newcastle Upon Tyne NE12 9TS, U.K.; § Fabric & Home Care Innovation Centre, The Procter & Gamble Company, Cincinnati, Ohio 45202, United States

## Abstract

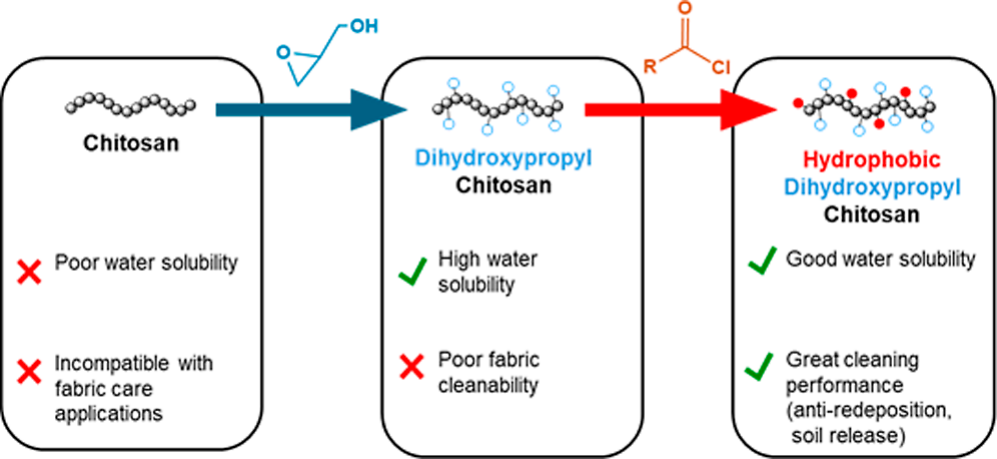

The design of more sustainable and eco-friendly solutions is one of the central challenges in the formulation of today’s laundry products. Water-soluble polymers are indispensable additives in laundry detergents as they play a wide range of functions. At present, the vast majority of these are still produced from petrochemical resources. In order to explore more sustainable alternatives, in this work, we have synthesized, characterized, and tested a novel group of anti-redeposition and soil release polymers based on hydrophobically modified 2, 3-dihydroxypropyl chitosan (DHPCH), a highly water-soluble chitosan derivative. Chitosan was selected on the basis of its environmental profile. Our results suggest that hydrophobic moieties are essential to observe cleaning benefits on synthetic based-textile. The level of modifications and the molecular weight of the unmodified chitosan were also shown to be decisive in conveying observable cleaning properties. This work is significant because it illustrates that DHPCH is a valid biorenewable platform for the development of new sustainable polymers for laundry detergents.

## Introduction

Water-soluble polymers are key ingredients in modern laundry detergent formulations. Their usage has mainly been driven by the soaring demand for innovative products in response to changes in consumers’ washing habits. Advancements in textile manufacturing technologies and compliance with more strict environmental cleaning regulations have also contributed to increased usage.^1,2^ Soil release polymers (SRPs) play an important role in laundry formulations. These are polymeric additives whose function is to promote the removal of soil adsorbed on textiles and prevent it from resettling during a washing cycle. Although their working mechanism is still not well understood, it has been shown that SRPs inhibit the adhesion of soil onto fibers by adsorbing onto the fibers surface and creating a hydrophilic layer.^3^

Traditionally, SRPs were developed to enhance the cleaning of synthetic fabrics, e.g., polyester (PE). On account of their high hydrophobicity, these fibers strongly interact with oily stains, thus making them difficult to remove. Copolymers of poly(ethylene terephthalate) and poly(oxyethylene terephthalate) (PET–POET) are often used in SRPs laundry detergent applications. This particular monomer composition makes them suitable for treating synthetic fabrics, such as PE. Indeed, while the hydrophobic terephthalate-containing moieties (that are chemically similar to PE textiles) drive the adsorption of the macromolecules onto the fabric surface, the hydrophilic polyoxyethylene-containing appendages stretch out toward the washing liquor, leading to the formation of an oil-repellent film that prevents soil from strongly interacting with synthetic fibers.^4^ Thermodynamically, the formation of a hydrophilic thin film reduces the water–fiber interfacial tension and makes the wetting of oily stains more difficult. Furthermore, the presence of SRPs on the fiber surface promotes the diffusion of water through the capillary spaces between the adsorbed oily stain and the fiber and enhance stain removal.^5,6^ Although PET/POET-based soil-release polymers effectively work with synthetic-based garments, they are mainly produced from petrochemical resources. The rising awareness around environmental problems, the irreversible consumption of fossil resources, as well as change in regulations are driving the development of more sustainable and environmental-friendly alternatives.^7^

Polysaccharides are natural polymers that have recently received great interest as starting materials for the design of novel laundry additives. Among these, chitosan has drawn great attention on account of its biocompatibility, versatility, and tunable chemical and physical properties.^8^ Chitosan is a natural copolymer characterized by two randomly distributed β-(1 → 4)-linked glucosamine units and residual *N*-acetyl glucosamine units. It is produced from the deacetylation of chitin, the latter being the second most abundant polysaccharide on Earth, and is mainly extracted from the exoskeleton of crustaceans, insects, algae and the cell wall of fungi.^9^ In contrast to the vast majority of polysaccharides that are soluble in water or other organic solvents, e.g., DMF, DMSO, and DMAc, chitosan is practically insoluble in any solvent (with the exception of water under certain conditions), thus making it more challenging to work with. Chitosan’s physical properties are directly related to its degree of deacetylation (DAC) and molecular weight. For instance, low-molecular-weight chitosan oligosaccharides exhibit high water-solubility under all pH conditions. Conversely, higher-molecular-weight chitosan samples (>10 kDa) are only soluble at pH < 6.5, where the amino groups are protonated.^10^ This protonation disrupts chitosan local chain-to-chain interactions and promotes water solvation.^11^ Although chitosan is soluble in water under mild acidic conditions, these are often not compatible with most applications, e.g., food, fabric and home care, skincare, medicine, etc. For this reason, several attempts have been made to enhance chitosan’s solubility under neutral or alkaline conditions. Many of these strategies require chemical modifications by taking advantage of chitosan’s two nucleophilic sites, i.e., its amino and hydroxyl groups, using chemistries such as alkylation, acylation, or Schiff base formation.^12,13^

In this work, we have synthesized 2, 3-dihydroxypropyl chitosan (DHPCH), a highly water-soluble chitosan derivative obtained by reacting chitosan with glycidol in acidic media.^14−16^ To endow DHPCH with specific physio-chemical properties and make it suitable for fabric care applications, libraries of modified DHPCH possessing a selection of compositions of hydrophobic appendages were synthesized and characterized and their cleaning properties (anti-redeposition and soil release) investigated. Structure–performance relationships in terms of molecular weight and hydrophobic moieties levels were constructed. It was found that the addition of hydrophobic appendages greatly enhanced the chitosan cleanability. This work suggests that DHPCH has potential as a platform for further development of biorenewable fabric care additives.

## Experimental Section

### Materials and Methods

Chitosan displaying a molecular weight of 700 kDa and a DAC of approximately 0.95 was acquired from Molekula Group. Chitosan displaying a molecular weight of 150 kDa and DAC of approximately 0.75 was acquired from Sigma-Aldrich. Chitosan displaying a molecular weight of 50 kDa and a DAC of approximately 0.88 was acquired from Glentham Life Science Ltd. Acetone (>99%), concentrated hydrochloric acid (37%), glycidol (Gly, 96%), benzoyl chloride, octanoyl chloride, 4-ethyl benzoyl chloride, triethylamine and dialysis membrane tubing (6–8 kDa MWCO) were purchased from Sigma-Aldrich. A base laundry detergent without SRPs was provided by P&G (Newcastle Innovation Centre). PE sheets loaded with BS2004 soil (SBL) were acquired from WFK Testgewebe GmbH. Polycotton stained squares were purchased from Accurate Product Development. All chemicals were used without further purification.

### Synthesis of Dihydroxypropyl Chitosan (**DC-50**)

A 500 mL round-bottom flask equipped with a magnetic stirrer and condenser tube was charged with chitosan (50 kDa, 5 g, 26.7 mmol) and an aqueous solution of hydrochloric acid (1% v/v, 265 mL). The solution was mixed at room temperature for 24 h to ensure that chitosan was completely dissolved. Then, glycidol (10.423 g, 140 mmol) dissolved in H_2_O (20 mL) was added to the chitosan solution dropwise using a syringe, and the reaction was stirred at 55 °C for 48 h under a nitrogen atmosphere. After cooling to room temperature, the reaction mixture was neutralized with a sodium hydroxide solution and precipitated in acetone (600 mL). The resulting crude product was collected via vacuum filtration, washed with acetone, and dried at 70 °C for 24 h under reduced pressure. DHPCH was dissolved in water (100 mL) and dialyzed against water for 3 days and then freeze-dried to afford a pale-orange solid (yield: 6.1023 g, 31.2%).

### Synthesis of O-Benzoylated Dihydroxypropyl Chitosan (**D50-B1h**)

A two-necked 100 mL round-bottom flask equipped with a magnetic stirrer and condenser was charged with **DC-50** (0.518 g, 2.1 mmol). After the DHPCH was completely dissolved, triethylamine (1.5 mL) was added to the solution in one portion. Then, the reaction mixture was placed in an ice bath for 20 min. Benzoyl chloride (0.7 mL, 6 mmol) was added dropwise to the reaction mixture via a syringe, and the mixture was stirred at 80 °C for 16 h under a nitrogen atmosphere. After cooling to room temperature, the reaction mixture was precipitated in 200 mL of acetone. The crude product was collected via vacuum filtration, dissolved in water (15 mL), and dialyzed against water for 3 d and then freeze-dried to afford a pale-yellow solid (yield: 0.13 g, 21.5%). Libraries of hydrophobic DHPCH derivatives were obtained by varying the amount and the type of acylating agent used. The list of all of the DHPCH derivatives prepared is reported in Table 3.

### Characterization

^1^H NMR, ^13^C NMR, and 2D (COSY and HSQC) spectra were recorded on a Bruker Avance 700 MHz spectrometer operating at 75 °C. Chitosan samples were prepared by dissolving 5 ∼ mg of the product in 600 μL of 1% DCl in D_2_O. DHPCH derivative samples were prepared by dissolving 5 mg of the product in 600 μL of D_2_O.

Quantitative ^13^C NMR spectra were acquired via an inverse-gated decoupling technique under the following conditions: concentration, 25 mg/mL; temperature, 75 °C; relaxation delay, 60 s; 3726 scans; internal standard TMS.

FTIR spectra (in the range of 400–4000 cm^–1^ wavenumbers) were recorded using an IR Affinity-1S Fourier transform infrared spectrophotometer equipped with an attenuated total reflectance (ATR) sampler at 4 cm^–1^ spectral resolution. For each experiment, 50 scans were recorded and averaged. Samples were dried overnight in a vacuum oven at 70 °C before each measurement.

### Soil Release Tests

Soil release tests were performed to evaluate the effect of textile surface modification by DHPCHs on the stain removal performance. Soil release tests were executed as reported in our previous work.^17,18^ PE fabrics were acquired from WFK Testgewebe GmbH. These were cut into 5 × 5 cm^2^ squares (tracers) and treated with DHPCHs solutions in an automatic tergotometer. In brief, stock solutions of DHPCHs were prepared in Milli-Q water (5% w/w). These were further diluted with hard water (7 gpg) into the tergotometer pots to a concentration of 12.5 ppm and mixed at 200 rpm for 10 min. Then, tracers were added and mixed thoroughly for 40 min at 30 °C followed by two 5 min rinse cycles. This procedure was repeated three times. Once collected, tracers were dried overnight under humidity and temperature control (50% RH, 20 ± 2 °C). Tracers’ weights were recorded at the end of the conditioning step (Wc). A negative reference leg was also included where tracers were preconditioned solely with water (no polymer addition).

Tracers treated with DHPCH solutions were stained with 200 μl of dirty motor oil (DMO) and then dried overnight. Once completely dried, tracers’ weights were recorded again (*W*_s_) to quantify the amount of DMO adsorbed (Δ*W*_1_). Stain removal tests were performed on an automatic tergotometer. Stained tracers were washed with a laundry detergent formulation for 40 min at 30 °C followed by two 5 min rinse cycles. Eight replicates were recorded for each experiment. Tracers were dried overnight under humidity and temperature control (50% RH, 20 ± 2 °C). Again, tracers’ weights were measured (Wr) to identify the amount of DMO removed (Δ*W*_2_). Soil release performance was monitored gravimetrically and via image analysis. Both methods were used to determine the stain removal index (SRI) values. With regard to image analysis, a reflection spectrophotometer (DigiEye) was used to acquire images of fabrics before and after washing against a white background. Images were analyzed using DigiEye software. For each tracer, the color of the motor oil stains was measured by reading the coordinates *L*_n_*, *a*_n_*, and *b*_n_* defined in the CIELAB color system of the stained area itself and the clean background fabric. From the measured coordinates, the differences in lightness (Δ*L*_n_*), redness (Δ*a*_n_*), and blueness (Δ*b*_n_*) in contrast to the unstained background area were calculated. The relative color changes, Δ*E*, were made to determine the level of staining compared to the unsoiled fabric using the equation below (eq 1), where suffix 1 denotes the values for the unsoiled background fabric prior to washing, and suffix 2 denotes the values for the stains. ΔE* was calculated for both unwashed (A) and washed stains (B).
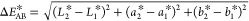
1

The SRI quantified via image analysis was calculated as follows using ΔE* values for unwashed stains (*A*) and washed stains (*B*)
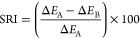
2

The SRI quantified gravimetrically was evaluated using the following equation
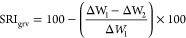
3where Δ*W*_1_ = *W*_s_ – *W*_c_ (the amount of stain adsorbed onto each PE fabric) and Δ*W*2 = *W*s – *W*r (the amount of stain removed after the final washing step).

### Anti-redeposition Test

Anti-redeposition tests were performed as described in a previous work.^17^ In brief, PE clean squares or tracers (5 × 5 cm^2^ garments) were washed under controlled conditions in an automatic tergotometer in the presence of a DHPCH solution and a typical laundry detergent formulation for four cycles. Each washing load was composed of four PE tracers; a certain number (31 or 21) of soiled ballast sheets (SBLs, 5 × 5 cm^2^ squares); and a sufficient number of knit cotton and poly cotton swatches to reproduce consumers’ washing loads with a total fabric concentration of 60 g/L. At the end of each washing cycle, consumed SBL squares were replaced with fresh ones. A new washing cycle was then performed. The same process was repeated four times. Finally, tracers were collected and dried under humidity and temperature control (50% RH, 20 ± 2 °C). In this work, three different anti-redeposition tests were performed in order to evaluate both the effect of DHPCHs chemical structure on the washing performance and the effect of the washing cycle conditions. In each test, a negative reference leg was included where fabrics were washed only with a laundry detergent containing no polymers. Test conditions are reported in Table 1.

**Table 1:** Summary of Anti-redeposition Test Conditions

test	polymer concentration (ppm)	laundry detergent concentration (ppm)	washing cycle parameters	number of stained swatches	water conditions	polymer tested
1	50	1950	40 min washing, 2·5 min rinse, 300 rpm	21	21 gpg 35 °C	DC-50-B1l DC-50-B1h DC-50-O1l DC-50-O1h DC-50 EB
2	50	1950	40 min washing, 2·5 min rinse, 300 rpm	31	21 gpg 35 °C	DC-50-B2l DC-50-B2m DC-50-B2h
3	50	1950	40 min washing, 2·5 min rinse, 300 rpm	31	21 gpg 35 °C	DC-150-B3l DC-150-B3h DC-700-B3l DC-700-B3h

The whiteness degree of the fabric tracers was then evaluated via image analysis, which was used to determine the whiteness index (WI), as defined by the International Commission on Illumination (CIE). A reflection spectrophotometer (Konica Minolta: CM-3630A) was used to obtain tracer images before and after the washing cycle. The color of each garment was monitored in terms of the coordinates *L*_n_*, *a*_n_*, and *b*_n_* defined in the CIELAB color system. The WI was calculated using the following equation (via the SpectraMagic NX software)
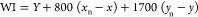
4where *Y* is the luminance factor, while *x* and *y* are the color coordinates of the observed garment defined in the Yxy color space. *X*_n_ and *y*_n_ are the color coordinates of the lighting source used (D65). Whiteness results were calculated as the difference (ΔWI) between the WI of tracers washed with DHPCH in the presence of the detergent formulation and the WI of tracers washed with the detergent formulation only.

## Results and Discussion

### Synthesis and Characterization

**Scheme 1:**
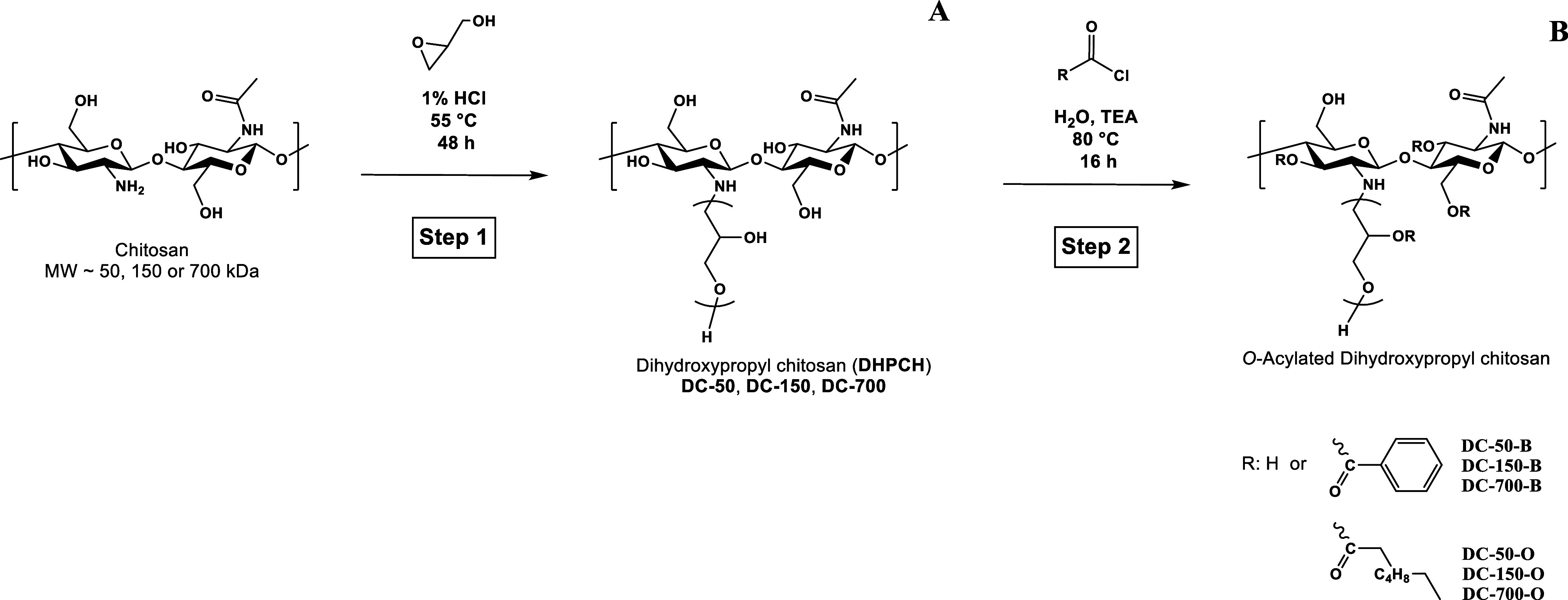
Synthesis of (A) Dihydroxypropyl Chitosan (**DC-50**) and (B) Hydrophobic Modified DHPCH

Although chitosan is soluble in water at pH 6.5 or below, acidic environments are often not compatible with fabric care applications (typical laundry detergents show a pH ranging from 7 to 11 when diluted under washing conditions). DHPCH is a highly water-soluble derivative that displays much improved solubility under all pH conditions and, therefore, is a superior platform for the development of novel chitosan-based laundry aids. The synthesis of DHPCH ethers was performed by reacting chitosan with glycidol in a mild acidic aqueous solution (Scheme 1, step1).^14−16^ The amino group on the C2 position and the hydroxyl groups on the C6 and C3 positions in each sugar unit of chitosan are likely to react with glycidol but at different rates, depending upon their nucleophilicity and steric hindrance.^19^ As will shortly be discussed, FTIR and NMR spectroscopic analysis of products confirmed that no reaction involved any of the hydroxyl groups, supporting the idea that the reaction occurred solely at nitrogen, presumably on account of its greater nucleophilicity. Furthermore, the grafting of glycidol moieties provides additional hydroxyl groups that can react with other molecules of glycidol, thus leading to the formation of poly(glycidol) oligomeric chains appended to chitosan.^20^ The structural properties of DHPCHs are defined by two parameters, namely, the degree of substitution (DS) and the average molar substitution (MS). The former defines the average number of substituted hydroxyl/amino groups per sugar unit, whereas the latter quantifies the average number of glycidol moieties per sugar unit.^20^ While the DS cannot be higher than 3, the MS has no theoretical upper limit. Our results indicated that typical MS values of 1.5 were obtained. Details concerning DHPCHs obtained from chitosan displaying various molecular weights and DACs are reported in Table 2. Overall, the synthetic method provided sufficient quantities of DHPCH derivatives with good levels of purity.

**Table 2:** Summary of Dihydroxypropyl Chitosan (DHCPH Structural Properties

product code			DHPCH			
	MW (kDa)	DAC^a^	DS^b^	MS^c^	yield (%)	chitosan/gly (mol/mol)^d^
DC-50	50	0.88	0.88	1.50	39.2	5.22
DC-150	150	0.75	0.75	1.40	39.4	5.23
DC-700	700	0.95	0.95	1.34	39.7	5.21

a Degree of deacetylation (DAC).

b Degree of substitution (DS), calculated by NMR spectroscopy.

c Average MS, calculated by NMR spectroscopy.

d Molar ratios between DHPCH and glycidol.

**Figure 1:**
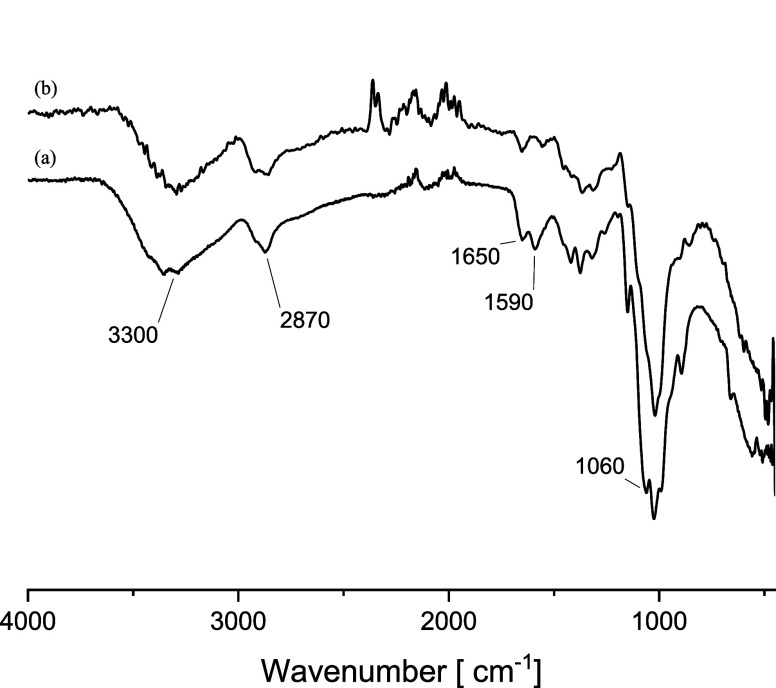
FTIR spectra of (a) unmodified chitosan and (b) dihydroxypropyl chitosan (**DC-50**).

The chemical structures of the DHPCH derivatives have been elucidated by means of FTIR, ^1^H NMR and ^13^C NMR spectroscopies. **DC-50** was selected as a representative of the DHPCH prepared in this study. FTIR spectra of **DC-50** and unmodified chitosan are shown in Figure 1. The transmittance profile of unmodified chitosan (Figure 1a) displays typical signals corresponding to functional groups of polysaccharides. The broad peak at ca. 3300 cm^–1^ arises from the stretching vibrations of N–H and O–H; the sharper absorption n band at 2870 cm^–1^ corresponds to the symmetric and asymmetric vibrations of saturated C–H; the peaks at about 1650 and 1590 cm^–1^ are associated with the stretching of the C=O of the secondary amide and the N–H bending of the primary amine, respectively.^21^ The grafting of glycidol moieties was confirmed (Figure 1b) by the complete disappearance of the signal at 1590 cm^–1^, suggesting that the hydrogen of the primary amino group was completely substituted by glycidol appendages.^14−16^

**Figure 2:**
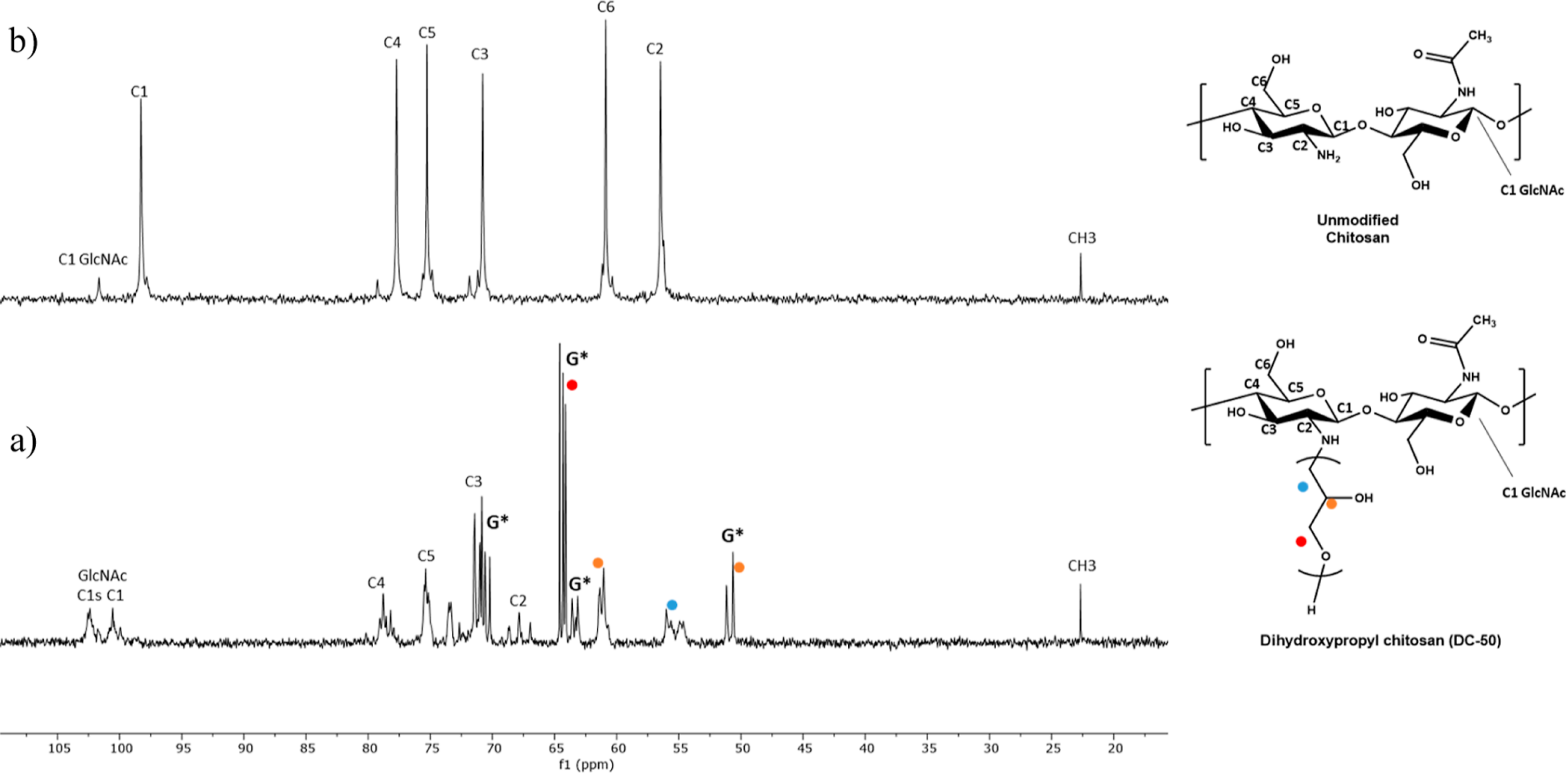
(a) ^1^H gated-decoupling ^13^C NMR spectrum (700 MHz, D_2_O) of dihydroxypropyl chitosan (**DC-50**), and (b) ^13^C NMR spectrum (700 MHz, D_2_O) of unmodified 50 kDa chitosan.

**Figure 3:**
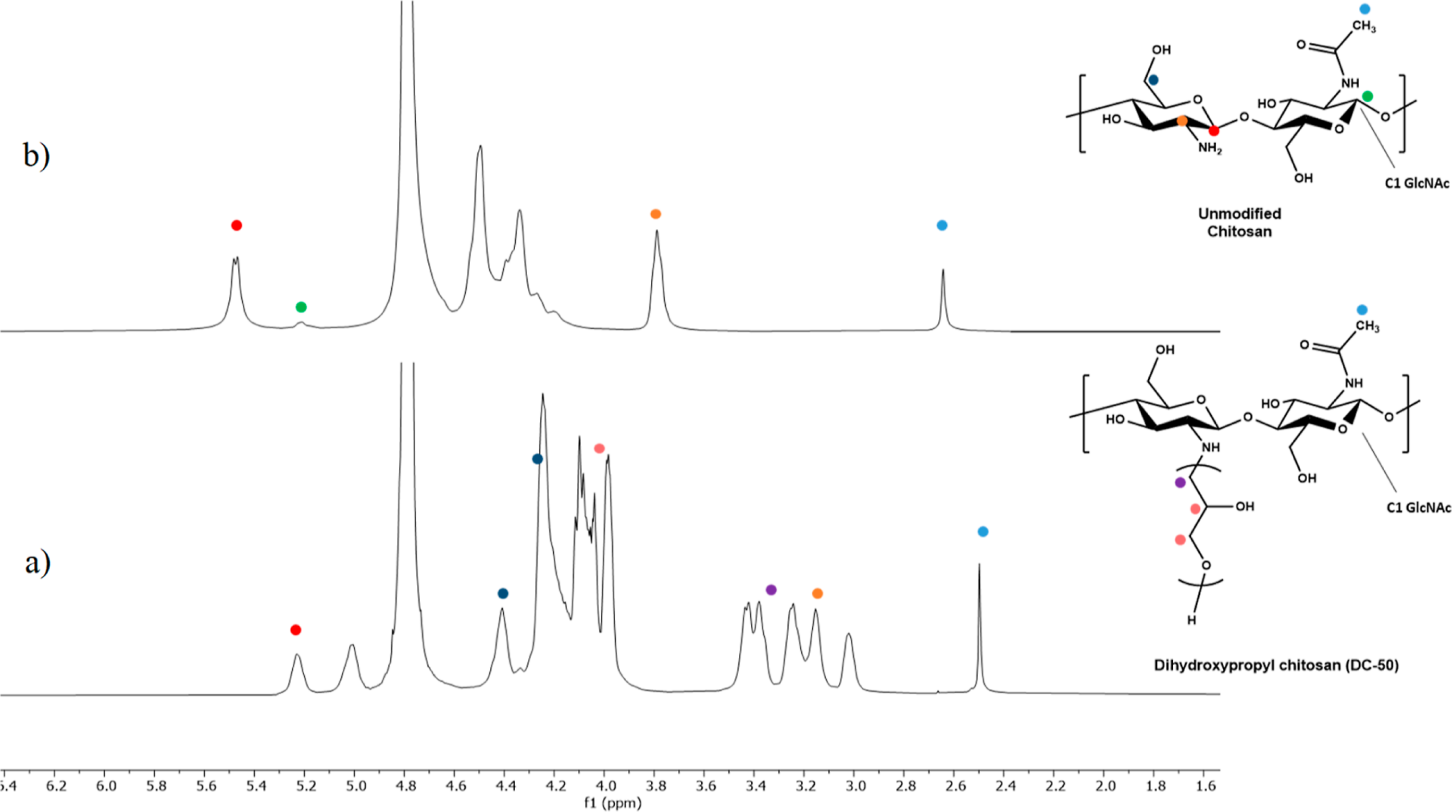
^1^H NMR spectra (700 MHz, D_2_O) of (a) dihydroxypropyl chitosan (**DC-50**) and (b) unmodified chitosan.

The ^13^C NMR and ^1^H NMR spectra of unmodified chitosan and **DC-50** are reported in Figures 2 and 3, respectively (2D COSY and HSQC NMR spectra of **DC-50** are available in the Supporting Information). The characterization of **DC-50** from the ^1^H NMR spectrum was challenging on account of the overlapping of the resonances associated with the dihydroxypropyl appendages and those arising from the protons of chitosan repeat units. Nevertheless, the use of 2D and quantitative ^13^C NMR spectra and their comparisons with dihydroxypropyl cellulose and polyglycidol from previously published^22−24^ works have allowed the resolution of the **DC-50** structure. Quantitative ^13^C NMR spectra were recorded via an inverse-gated decoupling sequence that minimizes the nuclear Overhauser effect, providing integrable carbon spectra.^25,26^ Focusing on Figure 2, multiple strong and sharp peaks, marked with the symbol G* in the spectrum of **DC-50**, were assigned to the highly mobile pendant appendages of glycidol grafted onto the more rigid backbone of chitosan. Among these, the collection of signals ranging from 64.7–64.3 ppm belongs to the O–**C**H_2_ methylene carbons of the glycidol moieties. The broader peak centered at 55.4 ppm is associated with the methylene carbon bonded to the amino group of chitosan (NH–**C**H_2_–). No variation was observed in the chemical shift of the signals associated with carbons C3 and C6, while the resonance of the C2 carbon shifted from 57.5 to 67.0 ppm, further suggesting that the amino group was exclusively involved in reacting with glycidol. The remaining sharp signals at 70.0 and 50.0 ppm were difficult to assign; however, we speculate that these arise from the resonance of methine carbons of the glycidol moieties. Based on these assignments, the DS was calculated by comparing the integration of the signal for the C-5 carbon (the latter integrated to 1 carbon) to that of the methylene group of glycidol bonded to the amino group of chitosan. The MS was quantified by comparing the integration of the signal for the C-5 carbon to that of methylene (O–**C**H_2_) of the glycidol units. The ^1^H NMR spectrum of **DC-50** is displayed in Figure 3a. The signals at 5.5 and 5.2 ppm were associated with the resonance of anomeric protons of the glucosamine and *N*-acetyl glucosamine repeating units of chitosan, respectively. The collection of signals ranging from 4.5 to 3.9 ppm can be assigned to H3, H4, H5, and H6 protons of the chitosan saccharide units. These overlap with the resonances of protons associated with the pendant glycidol moieties grafted onto the chitosan backbone. The signals at 3.0–3.5 ppm comprise the resonances of the chitosan H2 protons together with those ascribable to the remaining protons of bonded glycidol appendages. Lastly, the signal at 2.47 ppm appears sharp and corresponds to the three protons of the acetyl group of the chitosan *N*-acetyl glucosamine repeating units. Together, NMR and FTIR spectroscopic analysis suggest that the synthesized DHPCHs ethers are in agreement with the proposed structures and degrees of alkylation. Furthermore, no residual unreacted glycidol could be detected in the final products from both NMR and FTIR spectroscopic analysis.

**Figure 4:**
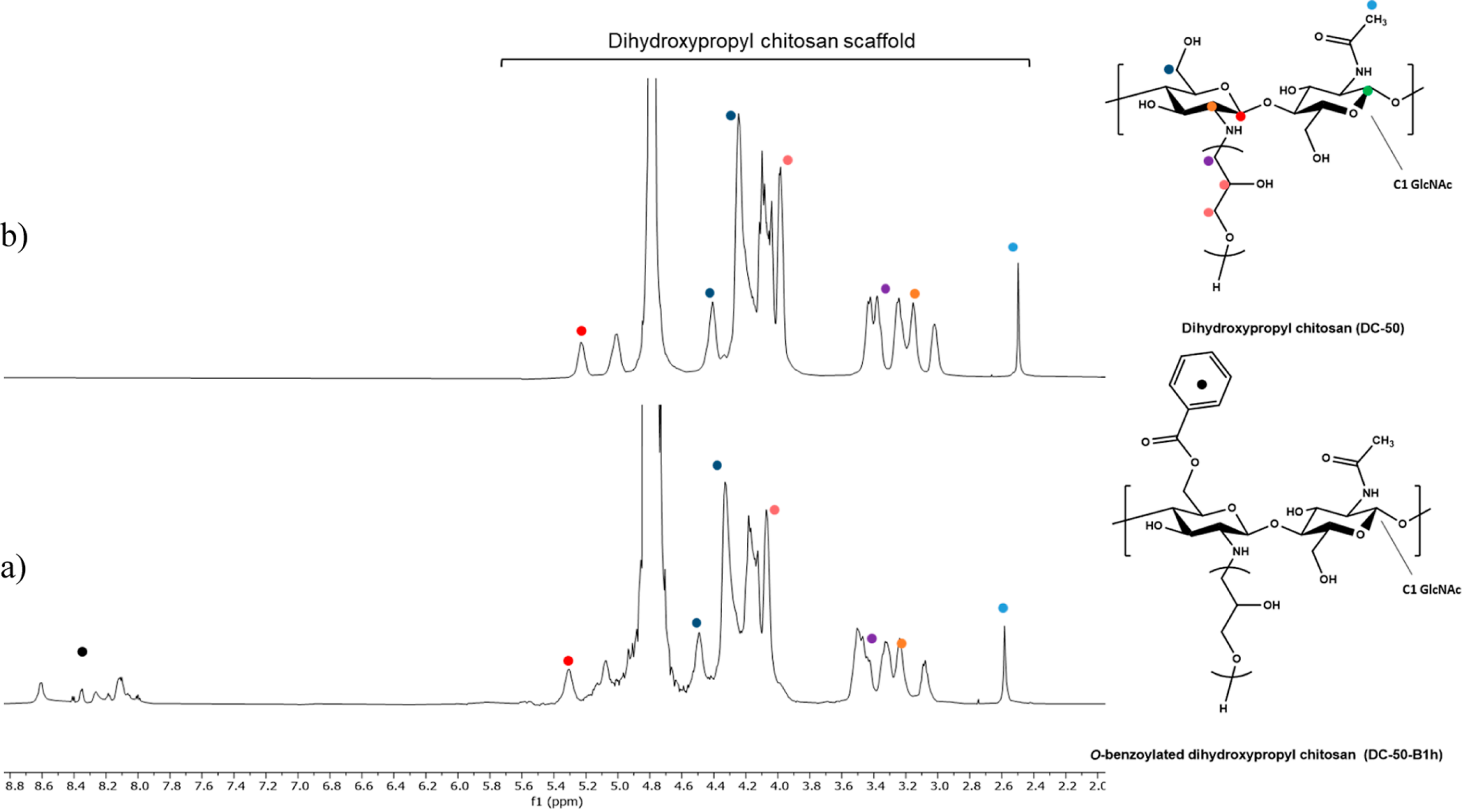
^1^H NMR spectra (700 MHz, D_2_O) of (a) hydrophobic modified dihydroxypropyl chitosan (**DC-50-B1h**) and (b) dihydroxypropyl chitosan (**DC-50**).

DHPCH is highly water-soluble, but it does not exhibit remarkable cleaning properties. For this reason, libraries of hydrophobic DHPCH derivatives were produced. Hydrophobization of hydrophilic polysaccharides has been shown to be crucial to tune their physicochemical properties in order to observe noticeable cleaning performance improvements.^17^ The decoration of DHPCH with hydrophobic appendages was performed in water by reacting DHPCH (synthesized starting from chitosan possessing three different molecular weights) with various acylating agents (benzoyl chloride, octanoyl chloride, and 4-ethyl benzoyl chloride) using triethylamine as a base (Scheme 1, step 2). The acylation can potentially occur at any of the chitosan hydroxyl groups or any of the hydroxyl groups of the glycidol moieties, thus leading to a random distribution of acylation upon the DHCPH scaffolds. Hydrophobically modified DHPCHs displaying a wide range of DS are reported in Table 3. Polymer **DC-50-B1h**, obtained by reacting **DC-50** with benzoyl chloride, was selected as a representative of hydrophobically modified DHPCH. The ^1^H NMR spectrum of **DC-50-B1h**, together with that of unmodified **DC-50**, is reported in Figure 4. Successful introduction of hydrophobic appendages was confirmed (Figure 4a) by the appearance of a new collection of signals between 8.0 and 8.7 ppm associated with the resonance of the five aromatic protons of the grafted moieties. No other significant variation from the spectrum of unmodified **DC-50** was detected. The DS of hydrophobic groups was quantified by using the signal of the acetyl group of chitosan *N*-acetyl glucosamine repeating units as a reference. Samples obtained from high-molecular-weight chitosan (**DC-150** and **DC-700**) exhibited high viscosity and were only moderately soluble in water, producing highly turbid solutions. Overall, our synthetic approach allowed the preparation of sufficient quantities of modified chitosans that were tested to assess their cleaning performance in a laundry formulation.

**Table 3:** Summary of DHPCH Derivatives Prepared via Acylation of DHPCH with Various Acyl Chlorides

product code^a^		hydrophobic dihydroxypropyl chitosan		
	MW (kDa)	DS^b^	yield (%)	DHPCH/hydrophobic (mol/mol)^c^
DC-50-B1l	50	0.01	11.8	1:2
DC-50-B1h	50	0.11	21.5	1:5
DC-50-O1l	50	0.02	21.0	1:1
DC-50-O1h	50	0.04	11.9	1:2
DC-50-B2l	50	0.02	25.6	1:2
DC-50-B2m	50	0.05	12.1	1:4
DC-50-B2h	50	0.18	10.8	1:6
DC-150-B3l	150	0.085	13.4	1:2
DC-150-B3h^d^	150	0.11	23.4	1:5
DC-700-B3l	700	0.07	11.6	1:2
DC-700-B3h^d^	700	0.1	34.4	1:5

a The numbers 1, 2, or 3 are used to differentiate between samples involved in the first, second, or third anti-redeposition test. **l**, **m**, or **h** indicate samples with low, medium, or high degrees of substitution, respectively. **B**, Benzoyl chloride; **O**, Octanoyl chloride.

b Degree of substitution (DS), calculated by NMR spectroscopy.

c Molar ratios between DHPCH and the acylating agents.

d Poor water solubility.

### Anti-redeposition Tests

**Figure 5:**
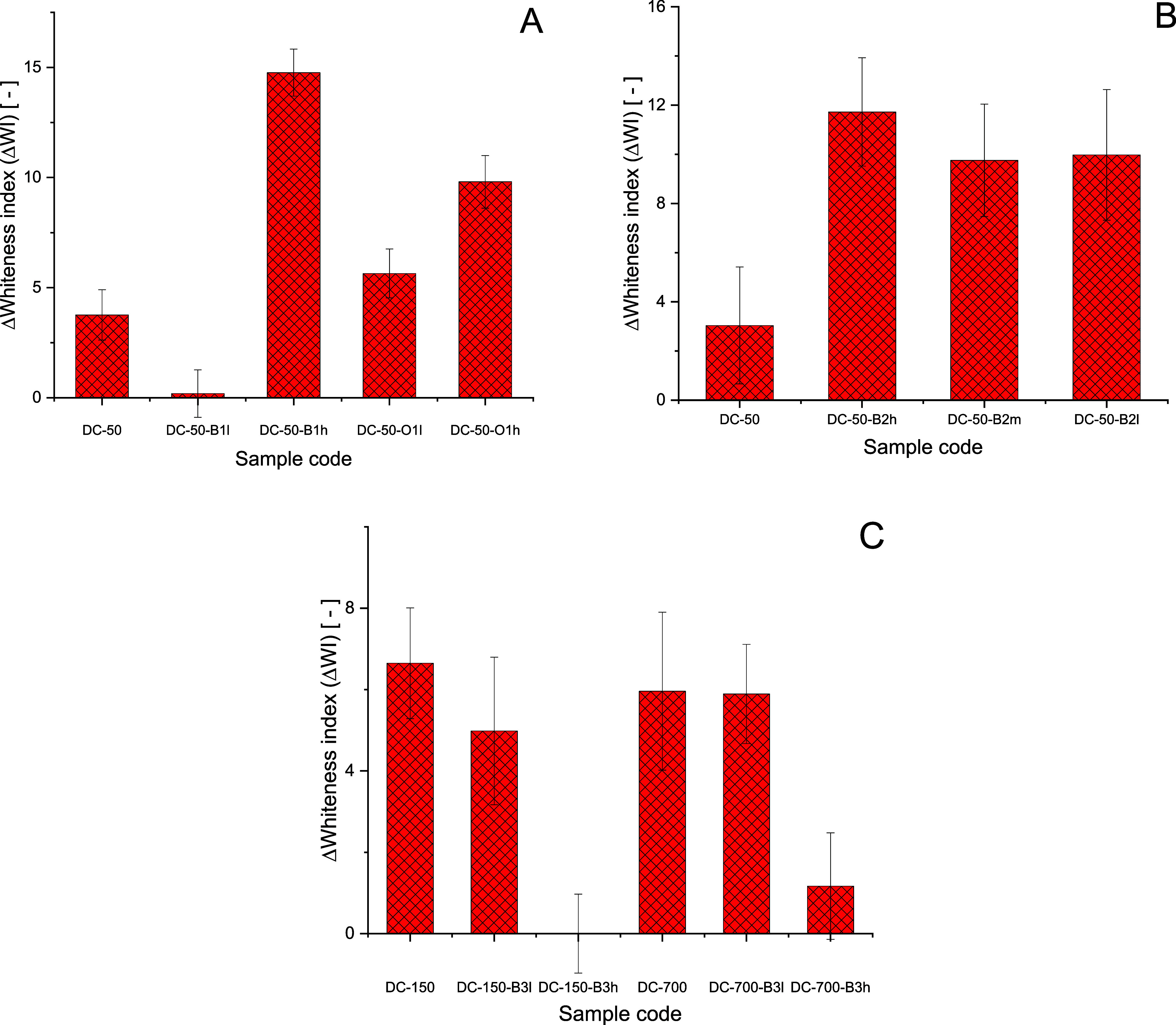
(A) Test 1 results: WI variation (ΔWI) of PE tracers washed with a laundry detergent formulation in the presence of DHPCH derivatives (**DC-50-B1h**, **DC-50-B1l**, **DC-50-O1h**, and **DC-50-O1l**). (B) Test 2 results: ΔWI of PE tracers washed with a laundry detergent formulation in the presence of DHPCH derivatives (**DC-50-B2h**, **DC-50-B2m**, and **DC-50-B2l**). (C) Test 3 results: ΔWI of PE tracers washed with a laundry detergent formulation in the presence of DHPCH derivatives (**DC-150-B3l**, **DC-150-B3h**, and **DC-700-B3l**, and **DC-700-B3h**).

Antiredeposition tests were performed to evaluate the ability of DHPCH derivatives to hinder the adsorption of soil onto garments and inhibit soil from resettling during multiple washing cycles, thus preventing textiles from showing loss of whiteness. Here, the cleanliness of DHPCH derivatives was assessed on PE fabrics. In an anti-redeposition test, clean swatches were washed with stained swatches under controlled conditions in the presence of an anti-redeposition aid. The cleanability of DHPCH derivatives was monitored by evaluating the whiteness degree (ΔWI) of washed fabrics via image analysis. Higher WI indexes are associated with better anti-redeposition performance.^17^ Three different tests were performed in order to evaluate the effect of (1) the modifying agent, (2) the washing conditions and composition of DHPCH derivatives, and (3) chitosan molecular weight. The results of test 1 are reported in Figure 5a. As anticipated, unmodified **DC-50** did not provide any remarkable performance. The grafting of hydrophobic moieties onto the chitosan backbone resulted in higher ΔWI values on PE fabrics. Specifically, the addition of aromatic (**DC-50-B1l** and **DC-50-B1h**) or aliphatic appendages (**DC-50-O1l** and **DC-50-O1h**) increased the anti-redeposition performance of unmodified DHPCH, providing superior whiteness levels. Overall, sample **DC-50-B1h** possessing the highest DS (0.11) offered the highest ΔWI values (more than 3-fold) on PE fabrics. Interestingly, a steady increase of the ΔWI values was observed when increasing the amount of grafted groups, suggesting that the hydrophobic modifications play a significant role in driving the cleaning performance of DHPCH derivatives.

A second test was performed in order to assess both the effect of the washing conditions and the composition of the DHPCH derivatives on the anti-redeposition performance. For the washing conditions, the effect of an increase (from 21 to 31) of the number of soiled swatches was monitored to simulate higher levels of stained clothes. Three different samples appended with benzoyl moieties (the best performing hydrophobic substituent from test 1) were tested to explore the effect of modifying the level of substitution (low, med, and high). Results of test 2 are reported in Figure 5b. Increasing the amount of stained swatches resulted in a reduction of the PE ΔWI values and flattening of the ΔWI variation among all tested samples. Indeed, despite their different DS, samples **DC-50-B2h** (DS = 0.18), **DC-50-B2m** (DS = 0.05), and **DC-50-B2l** (DS = 0.02) exhibited similar anti-redeposition performance, with sample **DC-50-B2h** performing slightly better than the others. This result is probably a consequence of the higher level of stains introduced in the test.

In our third test, the effect of chitosan molecular weight on soil anti-redeposition was investigated. The results of test 3 are reported in Figure 5c. Similar ΔWI values were observed for **DC-150**, **DC-700**, **DC-150-B3l** (DS = 0.085), and **DC-700-B3l** (DS = 0.07). Surprisingly, further increases of the DS values (**DC-150-B3h**, DS **=** 0.11 and **DC-700-B3h**, DS = 0.1) caused no effect on whiteness; this is probably a consequence of the poor solubility of higher-molecular-weight DHPCH derivatives.

Overall, these anti-redeposition tests confirmed that DHPCH derivatives are able to provide encouraging cleaning performance on synthetic textiles. Hydrophobic modification appears to greatly enhance DHPCH ability to prevent clean fabrics from graying during multiple washing cycles. Sample **DC-50-B1h** possessing the highest level of benzoyl appendages exhibited the best cleaning effect.

### Soil Release Test

**Figure 6:**
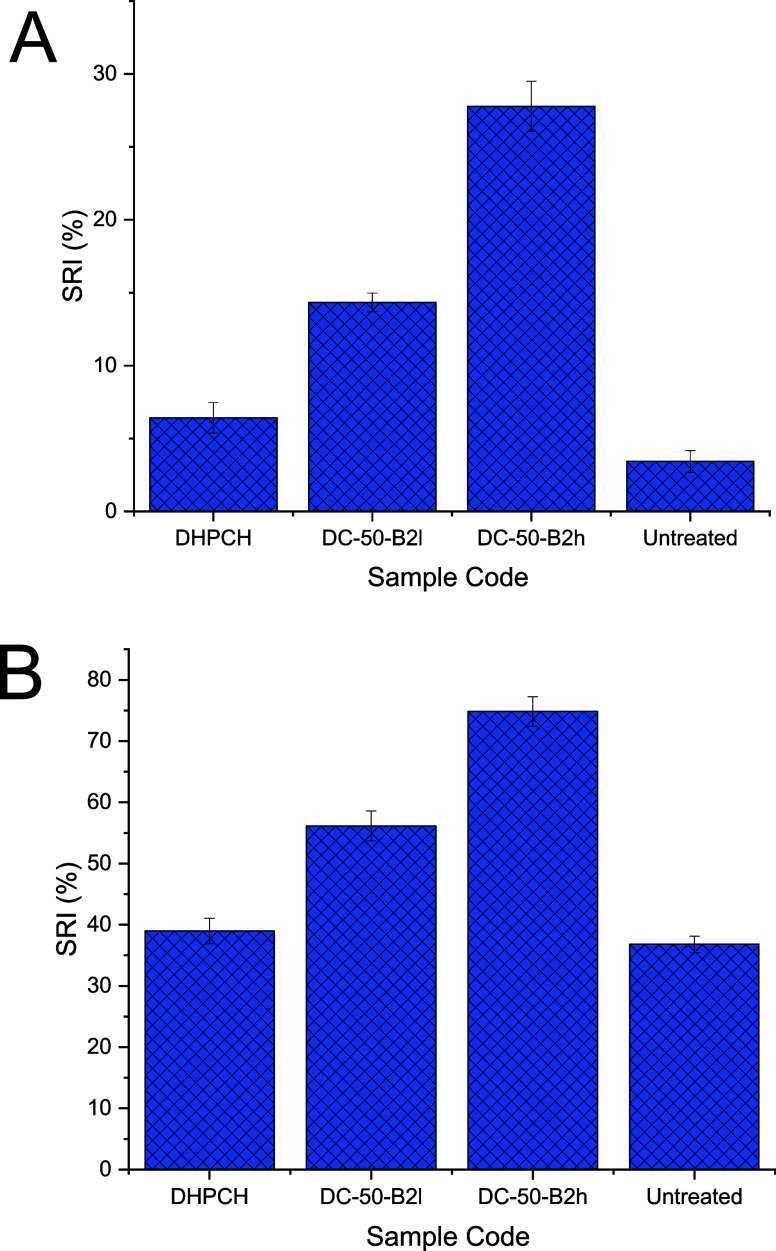
A) SRI evaluated via image analysis for PE fabrics pretreated with pure DHPCH and hydrophobic modified DHPCH (**DC-50-B2l** and **DC-50-B2h**). (B) SRI evaluated gravimetrically for PE fabrics pretreated with pure DHPCH and hydrophobic modified DHPCH (**DC-50-B2l** and **DC-50-B2h**).

Soil release tests were performed to assess the ability of DHPCH derivatives to modify PE fabrics, thus enhancing their soil removal properties. Soil release performance was evaluated in terms of soil release index (SRI) quantified both via image analysis (Figure 6a) and gravimetrically (Figure 6b). Samples **DC-50-B2l** and **DC-50-B2h** were selected as representative of hydrophobic modified DHPCH. PE fabrics treated with unmodified DHPCH (**DC-50**) exhibited SRI values similar to those of PE tracers washed solely with water (untreated). This is probably a result of a poor deposition of DHPCH onto synthetic fabrics. The introduction of hydrophobic appendages onto the DHPCH backbone resulted in a positive impact on soil release. Indeed, PE tracers conditioned with samples **DC-50-B2l** and **DC-50-B2h** displayed higher SRI values than PE tracers conditioned with sample **DC-50**, both via image analysis (Figure 6a) and gravimetrically (Figure 6b). Interestingly, higher levels of modifications were correlated to better soil release performance. Indeed, sample **DC-50-B2h,** which possesses the highest DS (0.18), displayed the highest SRI value on PE. This observation suggests that the addition of hydrophobic moieties positively affects the soil release efficiency of DHPCH on PE textiles, perhaps as a consequence of a better deposition of the hydrophobic modified polymers on PE. Both methods used to evaluate SRI performance revealed similar trends despite showing different absolute SRI values. On average, SRIs measured gravimetrically are higher than those quantified via image analysis. The visible dark staining effect of DMO is mainly due to several components such as carbon and carbon-based compounds produced via combustion, dust, and other contaminants or side products obtained from the breakdown of the oil itself. However, a large fraction of DMO is still represented by hydrocarbons and additives, which have a low visual staining effect. Once adsorbed on fabrics, these cannot be detected via image analysis and, therefore, lead to lower SRI values.

## Conclusions

In this work we have assessed the use of 2, 3-DHPCH derivatives as novel anti-redeposition and soil release aids for fabric care applications. The grafting of hydrophobic appendages was crucial to tune DHPCH physio-chemical properties and observe improvements in textile cleaning performance. Above all, the addition of aromatic moieties via acylation of DHPCH provided the best results in terms of soil anti-redeposition and soil release. The structure–performance relationship of DHPCH derivatives, in terms of chitosan molecular weight and hydrophobic content, was also evaluated. Our results clearly indicate that higher levels of hydrophobes are necessary to drive better anti-redeposition and soil release performance. Our findings provided new insights into the applicability of a relatively unexplored class of biorenewable polysaccharide in fabric care, highlighting the chemical features required in the design of novel laundry aids, and may encourage further development of chitosan in this application.

## Supporting Information Available

^1^H NMR spectra of hydrophobically modified 2, 3-dihydroxypropyl chitosans (PDF)
