# Supplementary material for: Dihydroxypropyl Chitosan: A Biorenewable Platform for the Design of Novel Fabric Care Additives
Source: Ind Eng Chem Res. 2024 Nov 27;63(49):21158–67. doi: 10.1021/acs.iecr.4c03632 (PMC11638941; doi:10.1021/acs.iecr.4c03632)
Supplement: Supplementary file 1 — ie4c03632_si_001.pdf [file ie4c03632_si_001.pdf]

Dihydroxypropyl chitosan (DHPCH): A biorenewable platform for the design of novel fabric care additives

Marcellino D'Avino[a], Ruth Chilton[b], Si Gang[b], Mark R. Sivik[c] and David A. Fulton[a]*

[a] Chemistry-School of Natural and Environmental Sciences, Newcastle University, Newcastle upon Tyne, NE1 8QB, United Kingdom

[b] The Procter & Gamble Company, Newcastle Innovation Centre, Newcastle upon Tyne, NE12 9TS, United Kingdom

[c] The Procter & Gamble Company, Fabric & Home Care Innovation Centre, Cincinnati, Ohio, 45202, United States

*Email david.fulton@ncl.ac.uk

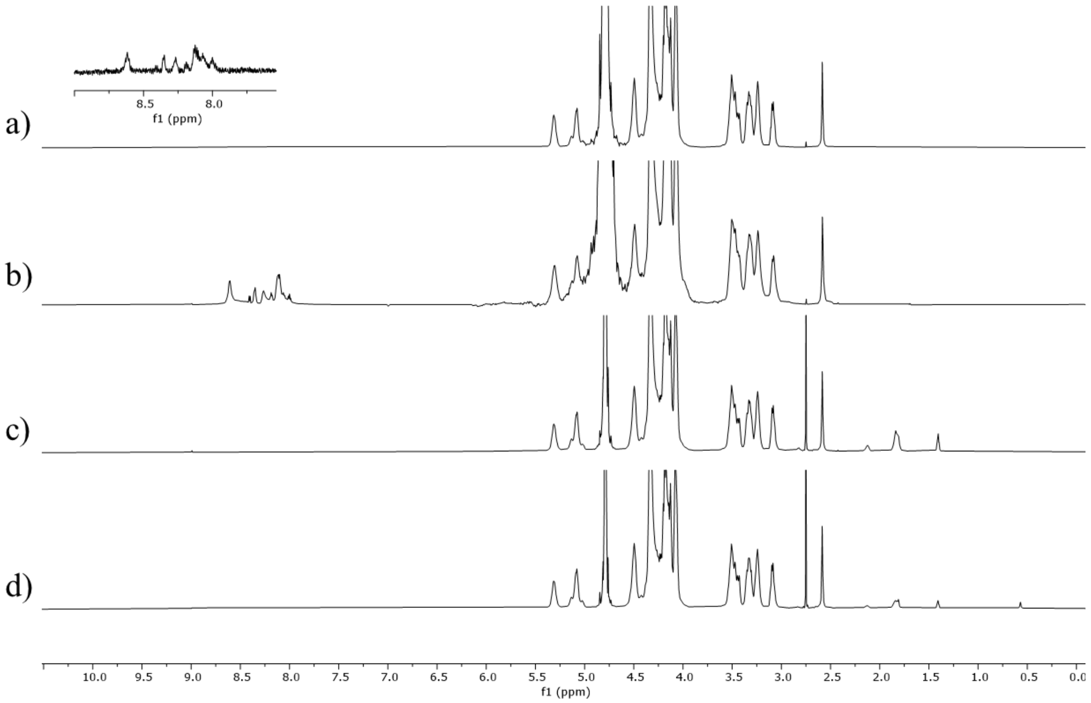

Figure S1: ^{1}H NMR spectra (700 MHz, D_2O) of (a) DC-50-B1l (b) DC-50-B1h (c) DC-50-O1h (d) DC-50-O1l

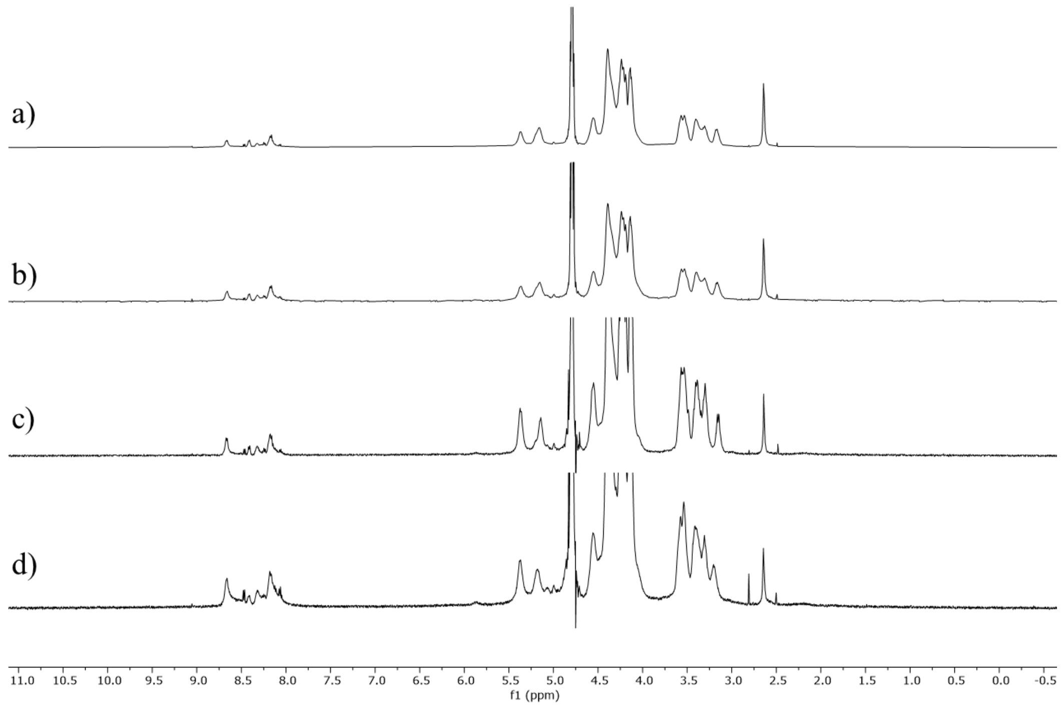

Figure S2: ^{1}H NMR spectra (700 MHz, D_2O) of (a) DC-150-B2l (b) DC-150-B2h (c) DC-700-B3l (d) DC-700-B3h

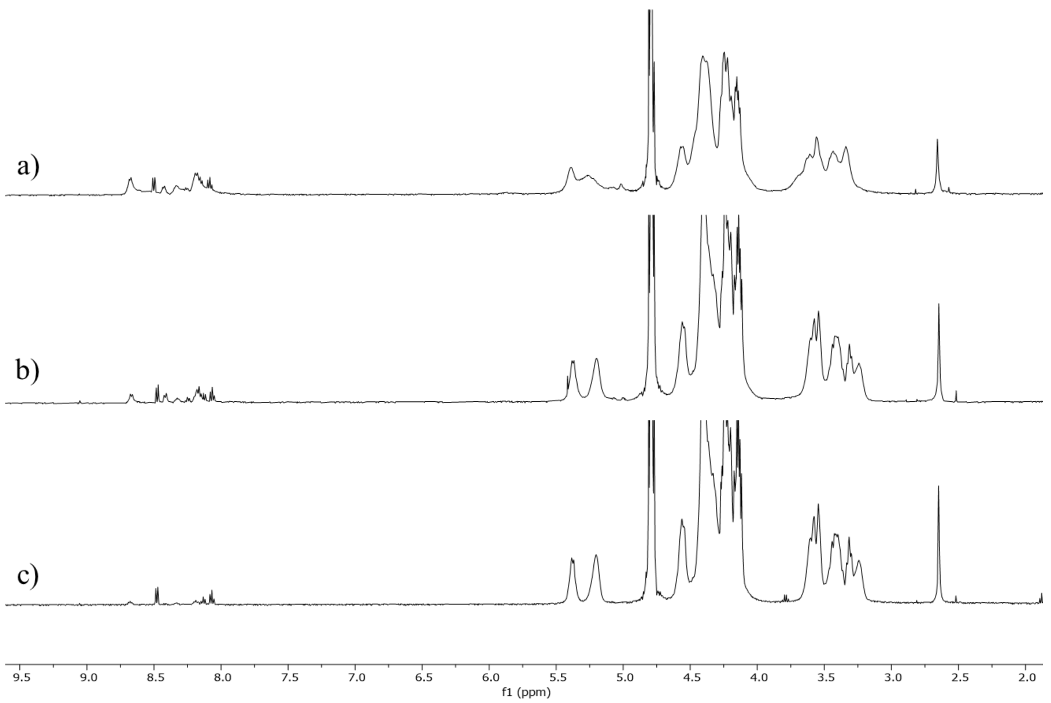

Figure S3: ^{1}H NMR spectra (700 MHz, D_2O) of (a) DC-50-B2h (b) DC-50-B2m (c) DC-50-B2l

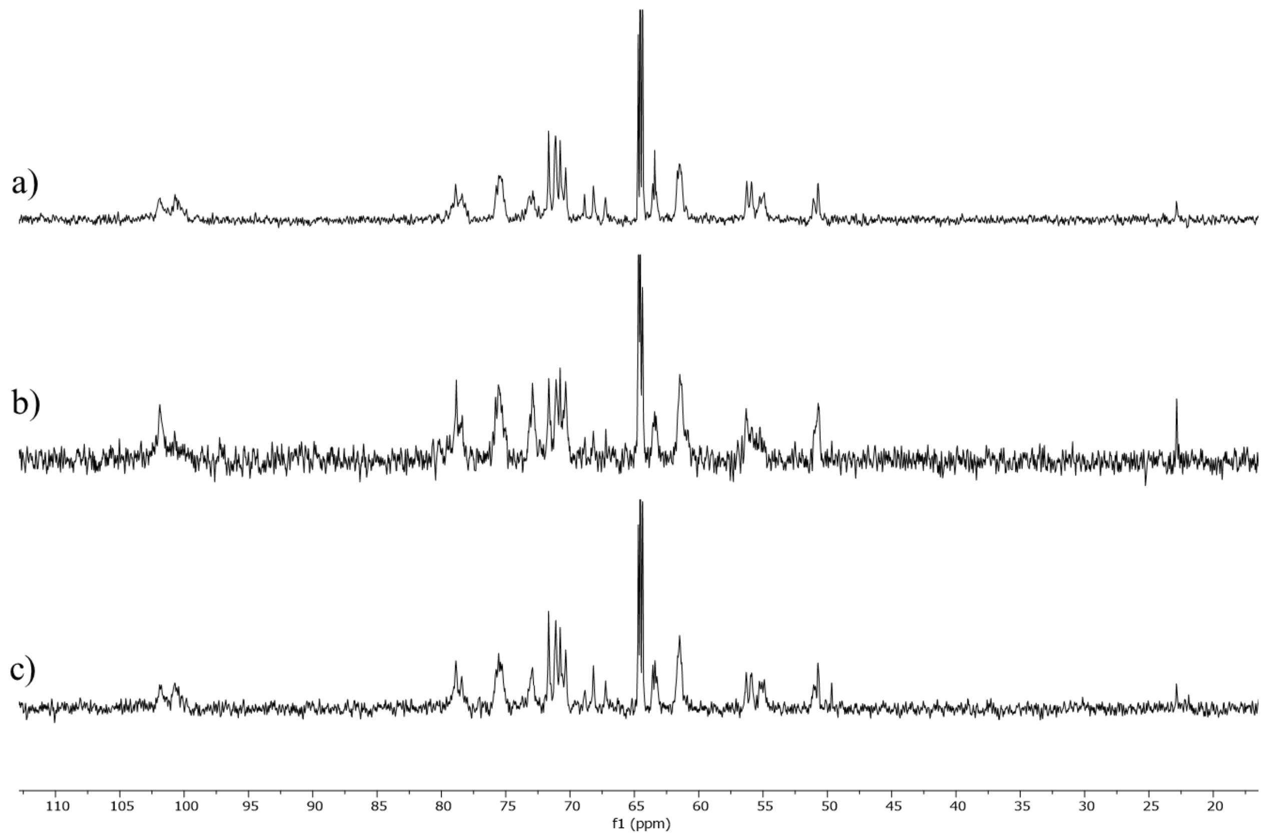

Figure S4: ^{13}C NMR spectra (700 MHz, D_2O) of (a) DC-50 (b) DC-150 (c) DC-700

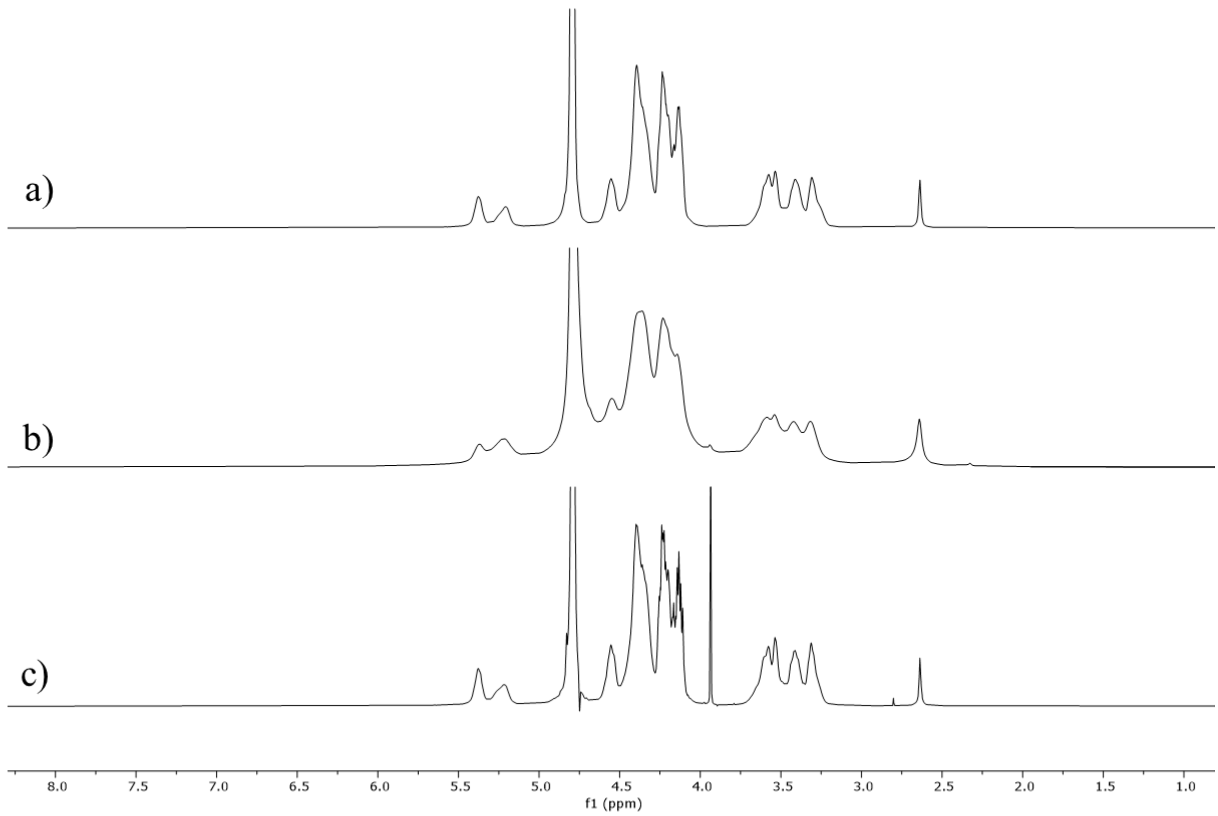

Figure S5: ^{1}H NMR spectra (700 MHz, D_2O) of (a) DC-50 (b) DC-150 (c) DC-700

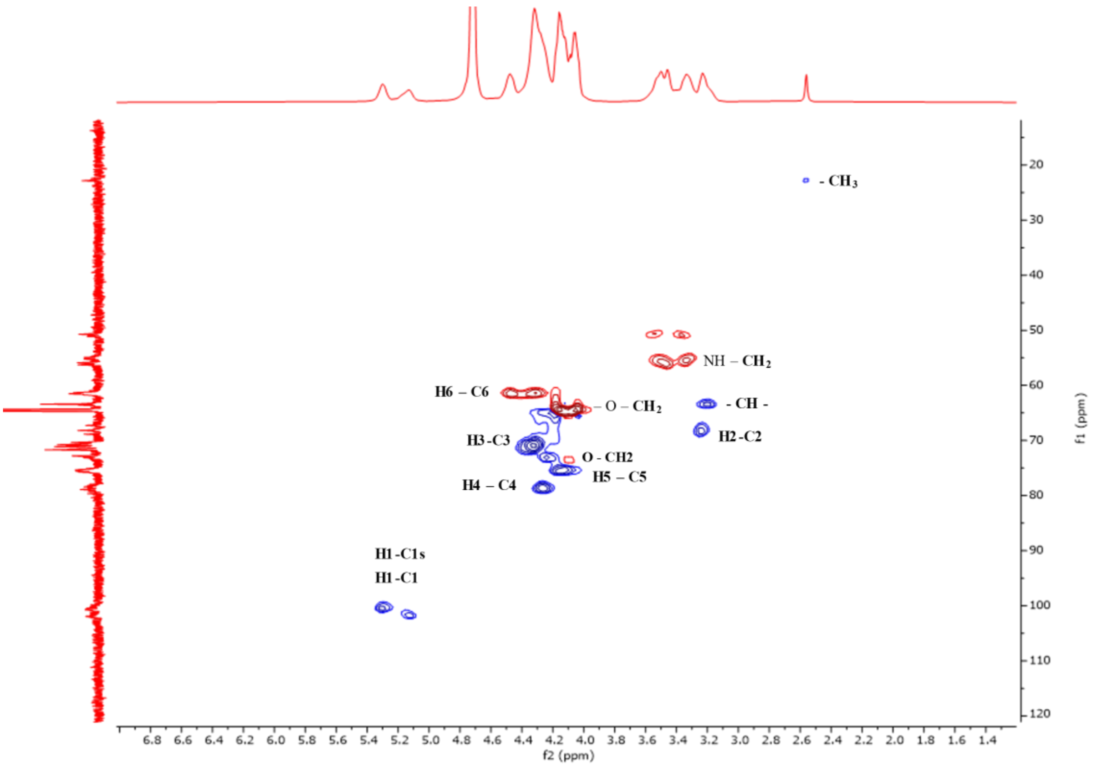

Figure S6: HSQC NMR spectrum (700 MHz, D_2O) of DC-50

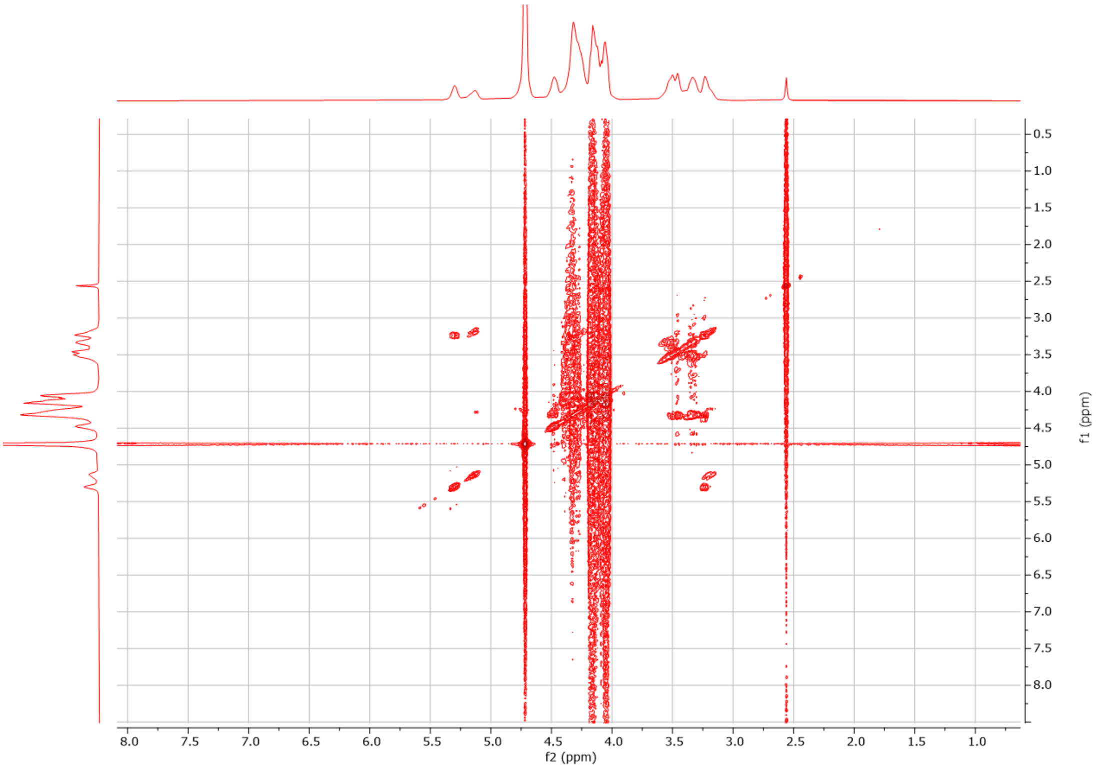

Figure S7: COSY NMR spectrum (700 MHz, D_2O) of DC-50
